# Serum metabolomics of treatment response in myasthenia gravis

**DOI:** 10.1371/journal.pone.0287654

**Published:** 2023-10-10

**Authors:** Patricia Sikorski, Yaoxiang Li, Mehar Cheema, Gil I. Wolfe, Linda L. Kusner, Inmaculada Aban, Henry J. Kaminski

**Affiliations:** 1 Department of Neurology & Rehabilitation Medicine, George Washington University, Washington, DC, United States of America; 2 Department of Pharmacology & Physiology, George Washington University, Washington, DC, United States of America; 3 Department of Oncology, Lombardi Comprehensive Cancer Center, Georgetown University Medical Center, Washington, DC, United States of America; 4 Department of Neurology, University at Buffalo/SUNY, Buffalo, New York, United States of America; 5 Department of Biostatistics, University of Alabama at Birmingham, Birmingham, Alabama, United States of America; University of California Riverside, UNITED STATES

## Abstract

**Objective:**

High-dose prednisone use, lasting several months or longer, is the primary initial therapy for myasthenia gravis (MG). Upwards of a third of patients do not respond to treatment. Currently no biomarkers can predict clinical responsiveness to corticosteroid treatment. We conducted a discovery-based study to identify treatment responsive biomarkers in MG using sera obtained at study entry to the thymectomy clinical trial (MGTX), an NIH-sponsored randomized, controlled study of thymectomy plus prednisone versus prednisone alone.

**Methods:**

We applied ultra-performance liquid chromatography coupled with electro-spray quadrupole time of flight mass spectrometry to obtain comparative serum metabolomic and lipidomic profiles at study entry to correlate with treatment response at 6 months. Treatment response was assessed using validated outcome measures of minimal manifestation status (MMS), MG-Activities of Daily Living (MG-ADL), Quantitative MG (QMG) score, or a strictly defined composite measure of response.

**Results:**

Increased serum levels of phospholipids were associated with treatment response as assessed by QMG, MMS, and the Responders classification, but all measures showed limited overlap in metabolomic profiles, in particular the MG-ADL. A panel including histidine, free fatty acid (13:0), γ-cholestenol and guanosine was highly predictive of the strictly defined treatment response measure. The AUC in Responders’ prediction for these markers was 0.90 irrespective of gender, age, thymectomy or baseline prednisone use. Pathway analysis suggests that xenobiotic metabolism could play a major role in treatment resistance. There was no association with outcome and gender, age, thymectomy or baseline prednisone use.

**Interpretation:**

We have defined a metabolomic and lipidomic profile that can now undergo validation as a treatment predictive marker for MG patients undergoing corticosteroid therapy. Metabolomic profiles of outcome measures had limited overlap consistent with their assessing distinct aspects of treatment response and supporting unique biological underpinning for each outcome measure. Interindividual variation in prednisone metabolism may be a determinate of how well patients respond to treatment.

## Introduction

Corticosteroids are an effective treatment for many autoimmune and inflammatory diseases; however, there is a significant minority of patients across conditions who respond poorly [1]. Identification of predictive markers of corticosteroid response would eliminate exposure to an ineffective therapy and spare patients from significant adverse effects [2]. Myasthenia gravis (MG) is a chronic autoimmune, neuromuscular disease caused primarily by antibodies directed towards the skeletal muscle nicotinic acetylcholine receptor [3]. For over fifty years high-dose prednisone has been a standard of care for MG, and retrospective studies demonstrate a large minority of patients have a poor response [4–7]. In 2016 the randomized, controlled MGTX clinical trial demonstrated the combination of thymectomy plus oral prednisone at high doses for months was superior to prednisone alone to improve strength and reduce disease severity for subjects with AChR antibodies [8]. A large minority of subjects within the study maintained high doses of prednisone and were symptomatic throughout the three-year trial regardless of whether they had received a thymectomy. As a rare, heterogenous disease, there are limited opportunities to perform rigorous discovery studies for biomarker evaluation. We took advantage of the MGTX biospecimen bank to perform a discovery study of markers that would predict a poor response to corticosteroids.

Metabolomics provides a powerful yet unbiased approach for identification of disease specific biomarkers of therapeutic responsiveness [9]. Metabolomic studies can assess therapeutic responsiveness from the perspective of global alterations in metabolism, which is ultimately dependent on specific disease pathophysiology, individual subject variation (influenced by genetics and environmental factors), and treatment. This approach has been successfully used to determine the phenotype of drug response in several diseases [10], predict radiation-induced cardiotoxicity [11], and detect pancreatic cancer in early stage [12]. Serum metabolomes differentiate between controls and patients with autoimmune diseases, including MG [13–15].

**Fig 1:**
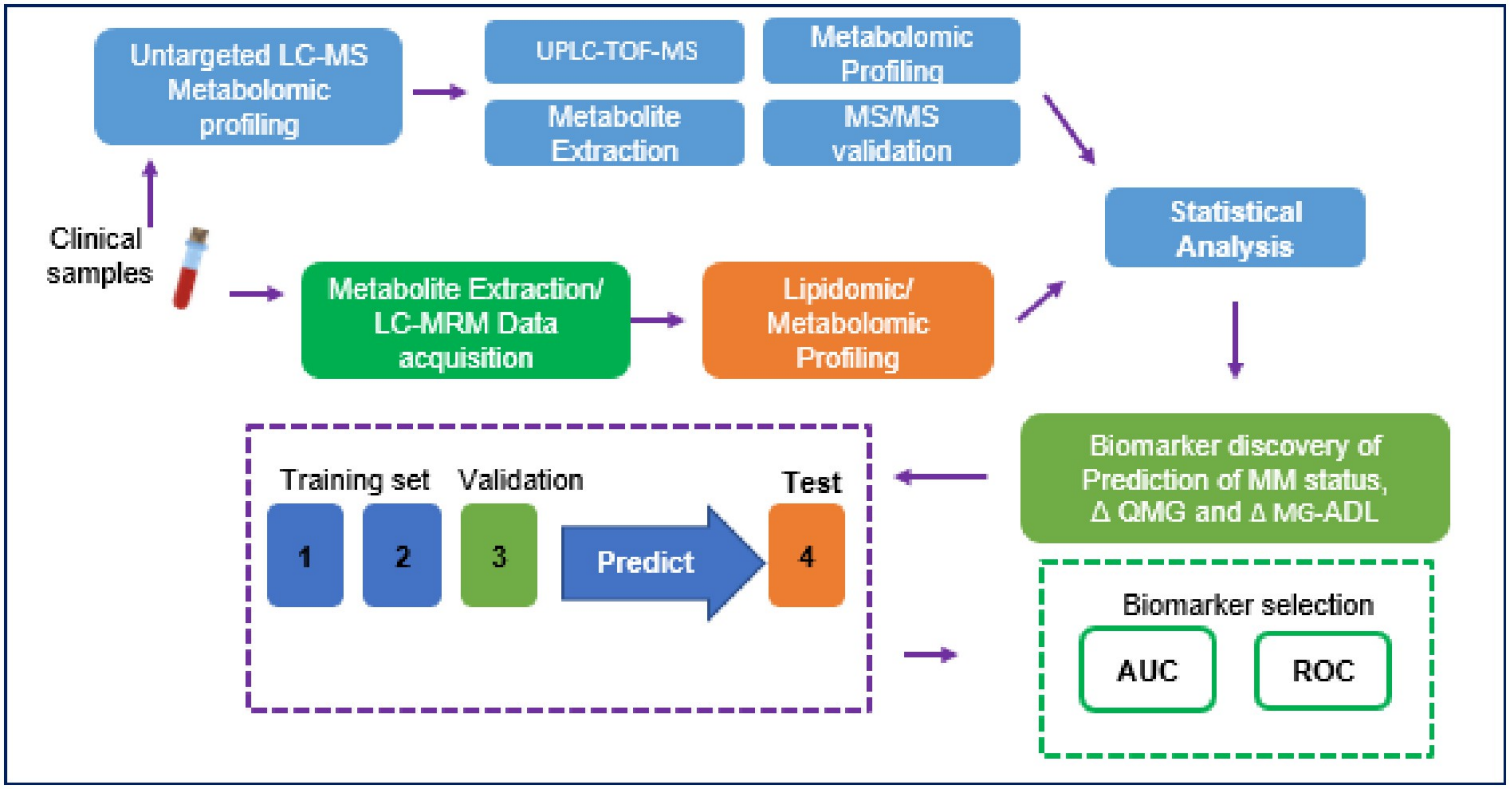
Workflow and data analysis pipeline for this study. Serum samples for 115 subjects from the MGTX study were prepared for targeted and untargeted LC-MS profiling. Statistical analyses and pathway analyses were performed to identify potential biomarkers.

Over the course of the three-year study, the MGTX clinical trial collected sera along with the Quantitative MG Score (QMG), MG-Activities of Daily Living (MG-ADL), and Minimal Manifestation Status (MMS). Major challenges for our investigation, and MG clinical research in general, is that the QMG and MG-ADL, despite validation and use as primary outcome measures, only show moderate correlation with each other [16]. In addition, the QMG is obtained at a single point evaluation of specific muscle strength, while the MG-ADL is a patient assessment considering performance of key activities over the previous one to two weeks [16, 17]. Therefore, we developed a categorization using MG-ADL and QMG to define high- and low-treatment responders in order to enhance the potential to identify treatment-responsive biomarkers. The specimen integrity and value of MGTX specimens have been confirmed through their successful use in several investigations [18–21]. The present experiments set out to test the hypothesis that a metabolomic or lipidomic signature at time of study entrance could be associated with a treatment response at 6 months. The overall study design is shown in Fig 1.

## Materials and methods

### Subjects

Sera were obtained over the course of the MGTX study (NS 42685). On day of collection blood was shipped by overnight delivery in chilled containers for immediate processing to the National Cancer Center in Frederick, Maryland where it was immediately processed to sera, which was maintained at -80° C until being thawed for metabolomic analysis. Subjects were between 18 and 65 years old with generalized MG for less than 5 years and elevated serum AChR antibody levels. All subjects received a standardized prednisone escalation and taper, which required subjects to have an improvement of QMG or a score less than 14. The prednisone dose over time (area under the curve analysis) at 6 months was not different between thymectomy plus prednisone versus prednisone alone groups [8].

### Clinical outcome measures

Initial subject stratification was based on three clinical outcome measures, which had been collected prospectively at 0 (baseline) and six months (M6, N = 92) over the course of the MGTX trial. The 6 month time point was used as a treatment response in the clinical trial was beginning to be seen and that a biomarker would be of greatest clinical value. Although subjects were treated with either thymectomy plus prednisone or prednisone alone, the groups were combined for the purpose of identification of common treatment responsive biomarkers. All analysis was performed in Excel and R environment. The MGTX study used designation of minimal manifestation status (MMS), the quantitative MG score (QMG), and the MG-activities of daily living (MG-ADL) to assess treatment response. We applied each of these measures to define treatment response as 1) MMS status obtained at 6 months, 2) QMG improvement of >40 percent from baseline vs. no response or worsening, and 3) MG-ADL >40% improvement vs. no improvement or worsening. Subjects with low to moderate improvement in QMG or ADL scores (1–40%) were not included in our analyses. We made these dichotomous definitions of extremely high and low responders with the expectation of a greater likelihood of discovery treatment-predictive markers. These treatment responses were then correlated with lipidomic and metabolomics results from baseline.

Only moderate correlation exists among outcome measures indicating that they measure different characteristics of MG severity [16]. Also, there were subjects who were highly responsive based on QMG, but not MG-ADL, and vice versa. Therefore, we developed a more selective definition of response combining MG-ADL and QMG criteria to better discriminate between responders and non-responders with the expectation that we would be more likely to identify a metabolomic signature predicting treatment response. We further defined subjects as treatment responsive vs. non-responsive by the algorithm: Responders (R) conditions 1 and 2 must be satisfied: QMG improve by > = 3 or > = 40% and MG-ADL improve by > = 2 or > = 40% or if ADL is 0 at baseline and M6, then only use QMG improvement criteria of > = 3 or > = 40%. The requirement of being > = 3 comes from previous studies which support a change in QMG score of 3 being clinically meaningful [22]. All non-responders (NR) had a QMG and MG-ADL that worsened or did not change unless the MG-ADL was 0 at baseline and M6.

### Metabolomics

This analysis quantitates around 270 endogenous small molecules using QTRAP^®^ 5500 LC-MS/MS System (Sciex). For this analysis, 25 μL of each serum sample was dissolved in 200 μL of extraction buffer (methanol/water 50/50) containing 200 ng/mL of debrisoquine (DBQ) as internal standard for positive mode and 200 ng/mL of 4-nitrobenzoic acid as internal standard for negative mode. The samples were vortexed for 30 seconds and incubated on ice for 20 min followed by incubation at -20 °C for 20 min. Samples were centrifuged at 13,000 rpm for 20 min at 4 °C. The supernatant was transferred to MS vial for LC-MS analysis. Five microliters of the prepared sample was injected onto a Kinetex 2.6 μm 100 Å 100 × 2.1 mm (Phenomenex) and resolved over 20 minute binary gradient. MRM transitions used for quantitation are detailed in S1 Table. Raw data were normalized to internal standard area and processed using MultiQuant 3.0.3 (Sciex). In order to ensure high quality and reproducibility of LC-MS data we conditioned the column using pooled QC samples followed by regular injections to monitor in the consistency of signal and retention time. We also used NIST plasma as standard reference material to monitor analytical variance. Blank solvent injections were performed after every 10 samples before and after pooled QC sample injections so as to monitor sample carryover. The report of pooled QC and NIST plasma is provided in S3 Fig.

### Deep lipidomics

This method is designed to measure 19 classes of lipid molecules which include diacylglycerols (DAG), cholesterol esters (CE), sphingomyelins (SM), phosphtatidylcholine (PC), triacylglycerols (TAG), free fatty acids (FFA), ceramides (CE), dihydroceramides (DCER), hexosylceramide (HCER), lactosylceramide (LCER), phosphatidylethanolamine (PE), lysophosphtatidylchloine (LPC), lysophosphatidylethnolamine (LPE), phosphatidic acid (PA), lysophosphatidic acid (LPA), phosphatidylinositol (PI), lysophosphotidylinositol (LPI), phosphatidylglycerol (PG) and phosphatidylserine (PS) using the QTRAP^®^ 5500 LC-MS/MS System (Sciex). For the analysis, 25 μL of each serum sample was extracted with 125 μL of chilled isopropanol containing internal standards for analysis by LC-MS/MS (S1 Table). Samples were chromatographically resolved using Xbridge amide 3.5 μm, 4.6 X 100 mm (water) using SIL-30 AC auto sampler online with QTRAP 5500 (Sciex, MA, USA) operating in positive and negative ion mode. A binary solvent comprising of acetonitrile/water 95/5 with 10 mM ammonium acetate as solvent A and acetonitrile/water 50/50 with 10 mM ammonium acetate as solvent B was used for the resolution using standard MS parameters. MRM transitions that were used for quantitation are detailed in S1 Table. The data were normalized to respective internal standard area for each class of lipid and processed using MultiQuant 3.0.3 (Sciex). The quality and reproducibility of LC-MS data were monitored rigorously as described in the previous section.

### Statistical analysis

Metabolomics and lipidomics raw data were filtered out if missing values > 20% or relative standard deviation for pooled QC samples (QC-RSD) >15%. All data were then normalized using the QC-RLSC algorithm (PMID: 21720319) to eliminate the analytical variance generated during data acquisition. All statistical analyses were conducted with SAS software (version 9.4; SAS Institute Inc., Cary; North Carolina, USA) and R (version 4.0.2). Potential group differences were tested using the non-parametric Mann Whitney U test. FDR correcting for multiple testing with Benjamini-Hochberg procedure two-sided for p-values < 0.05 were regarded as statistically significant. The prediction models were constructed and fitted using logistic regression. The performance of the biomarker prediction panel is evaluated using the AUC (Area under the ROC Curve).

### Study approval

The NINDS funded the trial and assembled an independent Data Safety Monitoring Board. Sites received local institutional review board (IRB)/ethics committee approvals, and each patient provided written informed consent before study entry including provision of serum samples. All specimens were deidentified. The George Washington University IRB provided additional review and approved these investigations.

## Results

### Clinical outcome measures define treatment unresponsive subjects

**Fig 2:**
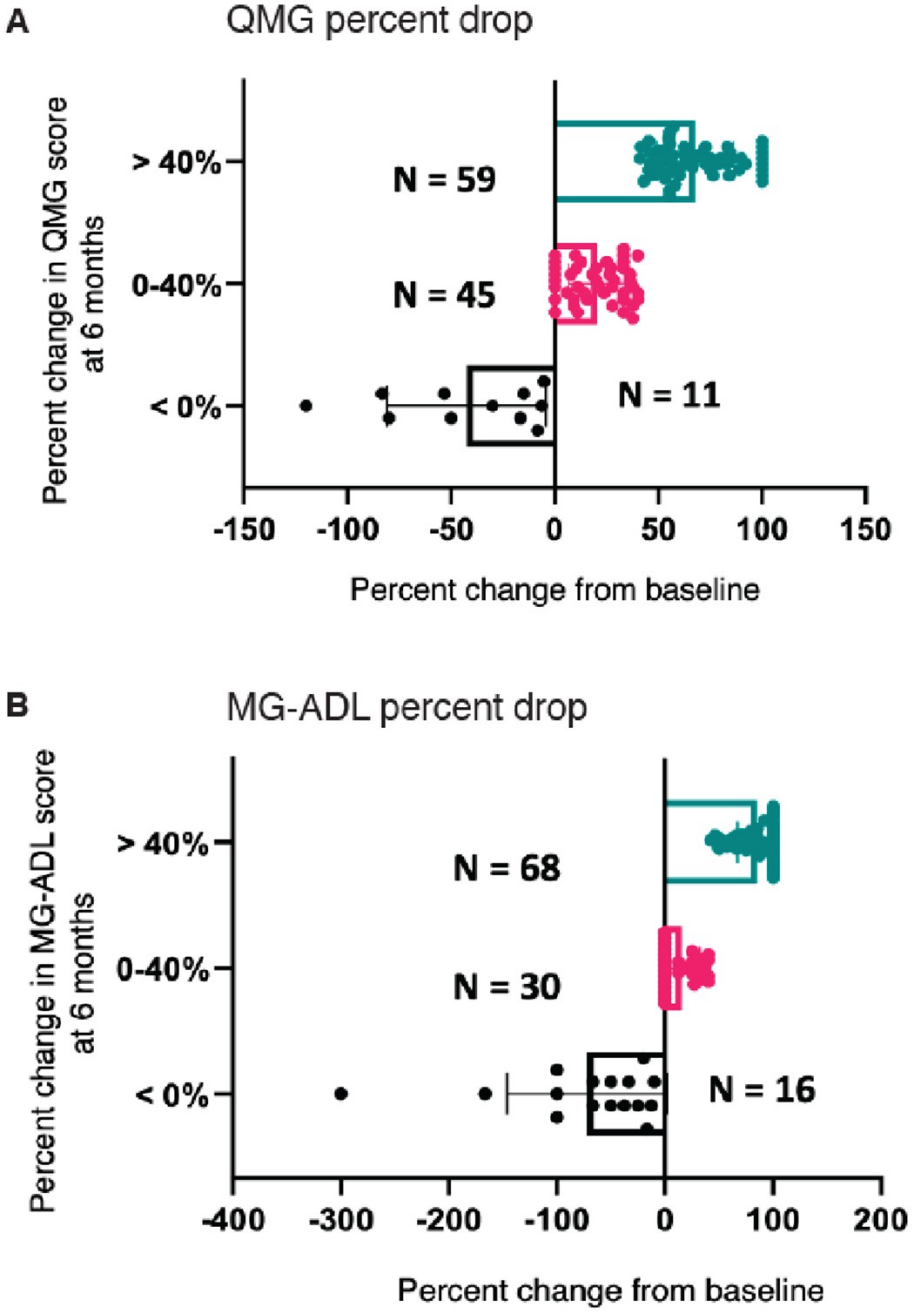
Percent drop of (A) QMG score and (B) MG-ADL score from baseline to 6 months post treatment. Subjects are grouped according to percent change in score (> 40% (represented as teal dots), 0–40% (represented as pink dots), or < 0% (represented as black dots)). Subjects with score > 40% improvement in QMG and MG-ADL are defined as treatment responsive whereas those with < 0% (no improvement or worsening score) are defined as treatment non-responsive.

The MGTX study was an international, multi-center study of AChR antibody-positive MG patients age 18 to 65 years with less than 5 years of disease duration, designed to assess the potential added benefit of thymectomy to prednisone [8]. Subjects were randomized to receive thymectomy plus prednisone or prednisone alone with a standardized escalation of prednisone dose to 100 mg every other day or 1.5 mg/kg body weight, whichever was lower. Table 1 shows the characteristics of subjects who had serum samples available at study entry (baseline). We used achievement of minimal manifestation status (MMS) at 6 months as a binary outcome measure. Of 115 total subjects, sixty-one subjects had achieved MMS at 6 months. Fig 2 shows the distribution of QMG and MG-ADL grouped as described in the Methods. Eleven subjects had a worsening of QMG (increased score) and another eight had no change. Fourteen subjects had an improvement of 0–20%, another 23 improved by 21–40% Fifty-nine subjects improved by >40%, which was used as our comparison group to those with no improvement or worsening. Seventeen subjects showed a worsening in MG-ADL score. Sixty-eight subjects showed a greater than 40% improvement in MG-ADL scores and were compared to the no improvement or worsened group. A general concordance was present between QMG and MG-ADL categorization; however, a lack of concordance was found in the low response groups as follows: < 0% (N = 5), no change (N = 2), 0–20% (no overlap) and 20 to 40% (N = 2). In contrast, an overwhelming majority of patients (N = 49) overlapped between the MG-ADL and QMG groups in those that showed > 40% improvement in either of these outcomes.

**Table 1:** Clinical cohort statistical characteristics of MM status, QMG scores and MG-ADL scores.

N = 115	Mean	SD	Range
**Age**	34.74 years	12.59	18–63
**Women**	80 (70%)		
**Race/Ethnicity**			
	Asian = 5 (4%) Black/African American = 10 (9%) White, not Hispanic origin = 58 (50%) Hispanic 34 (30%) Other (Mixed/Native American Alaskan) = 8 (7%)		
**Disease Duration**	1.44 years	1.04	0.02–4.41
**Prednisone at baseline**	47.87	34.91	0–120
**On Prednisone at entry**	89 (77%)		
**Thymectomy**	61 (53%)		
**MMS at 6 months**	51		
**QMG at baseline**	11.8	5.1	1–24
**QMG at 6 months**	7.3	5.0	0–23
**ADL at baseline**	5.2	3.3	0–13
**ADL at 6 months**	2.6	2.9	0–12

Using the criteria described in the Methods, 69 subjects were defined as responsive, 11 non-responsive and 23 were not categorized. The purpose of this approach was to establish the most rigorous definition of treatment-response to increase the likelihood of discovering treatment-responsive biomarkers. Intermediate levels (1–40% improvement in ADL or QMG score) of response were excluded from the final analysis. Based on the initial review of outcome measures, a dichotomous categorization was developed to define responsive or non-responsive sub-cohorts as described in the Methods, and were used to inform predictive biomarker discovery. No differences were identified in subjects classified into any of the responsive categories based on age, gender, receipt of thymectomy, or prednisone treatment at baseline. Therefore, we were able to perform our analysis regardless of whether patients had received a thymectomy or were receiving corticosteroids at study entry. Three patients in the non-responsive group during the first 6 months of the study received intravenous immunoglobulin, one at month 1 and two at month 4. No one in the non-responsive group received plasma exchange in the first 6 months of the study.

### Metabolomic and lipidomic analyses

One hundred and fifteen baseline serum samples (Table 1) were subject to targeted and untargeted metabolic and lipidomic profiling using ultra high-performance liquid chromatography coupled with time-of-flight mass spectrometry. This untargeted analysis yielded a total of 5343 metabolic and lipidomic targets that were subjected to statistical analyses to delineate a panel of potential predictive biomarkers using the established clinical outcome measures. For targeted analysis we identified 241 metabolites and 785 lipids.

### Metabolite and lipid profiles associate with clinical outcome

**Fig 3:**
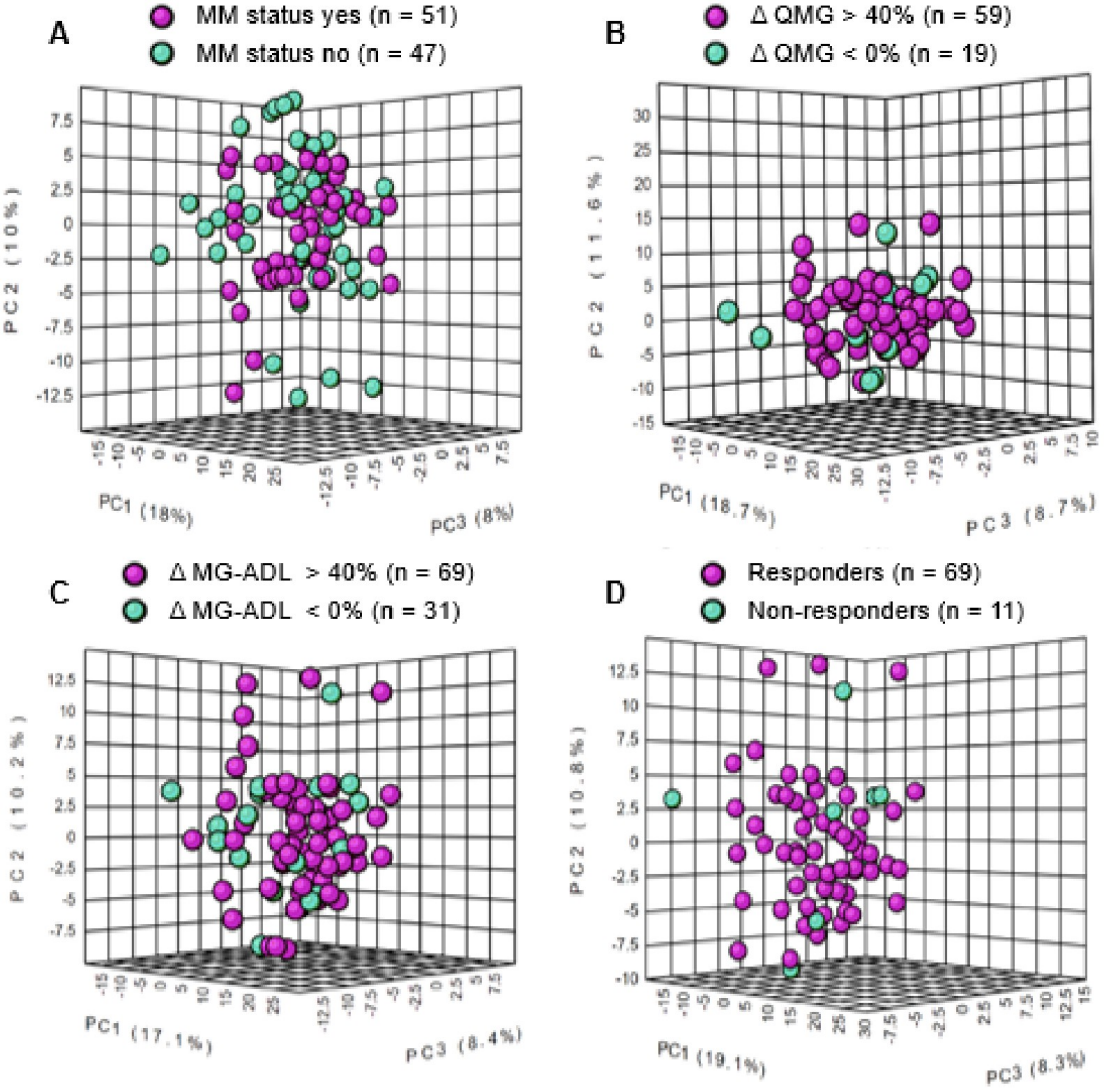
A three-dimensional plot showing metabolomic data structure for subjects in each clinical outcome measure. Principal component analysis (PCA) shows intergroup separation is shown on the X axis and intra-group variability on the Y axis based on serum metabolite and lipid profiles. Treatment responsive subjects are represented in cyan blue and treatment non-responsive subjects are represented in purple in each plot. (**A**) MM Status (Yes n = 51 vs. No n = 47) (**B**) change in (Δ) QMG score (> 40% n = 59 vs ≤ 0% n = 19), (**C**) change in (Δ) MG-ADL score (> 40% n = 69 vs < 0% n = 31), and (**D**) Responders (Responders n = 69 vs Non-responders n = 11).

We examined the global data structure of the metabolic and lipidomic profiling data using principle component analysis (PCA) of untargeted features for each outcome measure at baseline that resulted in reasonable interclass separation based on each of the clinical outcome measures (Fig 3). The PCA analysis demonstrates separation of each of the dichotomous classifications for each outcome measure. MMS shows less separation compared to the other PCA plots using the other outcome measures.

**Fig 4:**
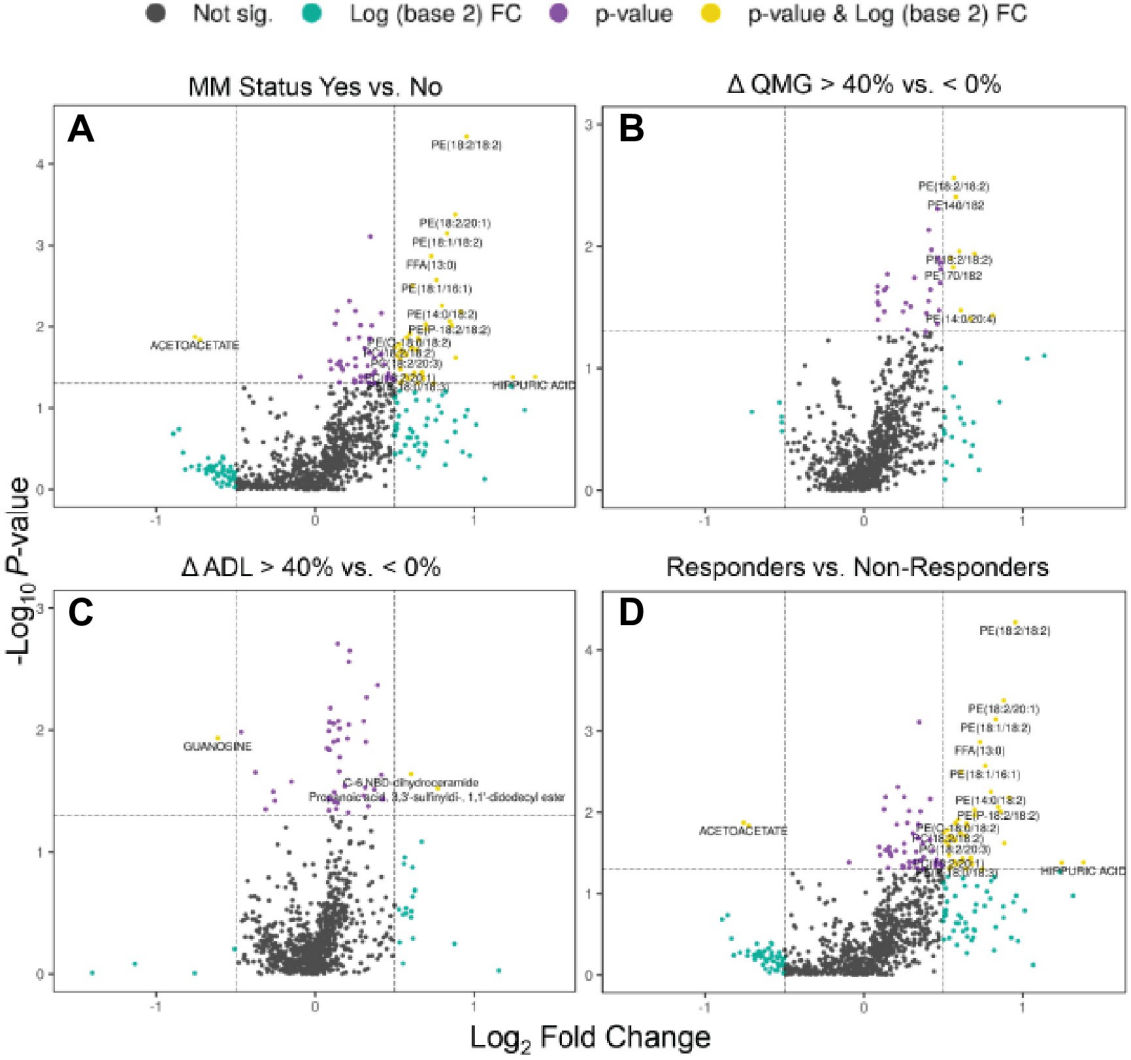
Volcano plots showing comparisons of serum profiles between treatment responsive and treatment non-responsive subjects for each clinical outcome group. (**A**) MMS status (responsive Yes vs non-responsive No) (**B**) change in (Δ) QMG (responsive > 40% vs. non-responsive ≤ 0%), (**C**) change in (Δ) MG-ADL (responsive > 40% vs. non-responsive ≤ 0%), and (**D**) Responders vs Non-Responders. Gray dots represent metabolites and lipids that were not significantly altered between treatment responsive and non-responsive subjects, teal dots represent metabolites with a fold change greater than 2-fold (cutoff), purple dots represent metabolites and lipids with a significant p value (p < 0.05) and yellow dots indicates those metabolites and lipids with both a significant fold change and p-value. Differences between outcome groups were tested using the non-parametric Mann Whitney U test and FDR correcting for multiple testing with Benjamini-Hochberg procedure two-sided. P-values < 0.05 were regarded as statistically significant. Individual p-values for each metabolite and lipid are found in S2 Table.

Differentially abundant metabolites and lipids between the outcome measures were visualized using volcano plots (Fig 4). Metabolites were selected based on significant p-values (X-axis) and fold change (Y-axis) and are represented as red dots while the non-significant metabolites are represented in black. The blue dots represent metabolites with significant differences (P<0.05). The full list of annotated metabolites and lipids can be found in S1 Table and statistical analyses for each metabolite and lipid can be found in S2 Table. The plots using MMS (Fig 4A), change in QMG (Fig 4B), and the Responders (Fig 4D) classification are similar with an overall upregulation of phospholipids, while the change in MG-ADL plot (Fig 4C) shows a different pattern of metabolite abundance. This suggests that despite the combined use of QMG and MG-ADL for responder definition, the metabolic and lipidomic signatures are driven by change in QMG.

**Fig 5:**
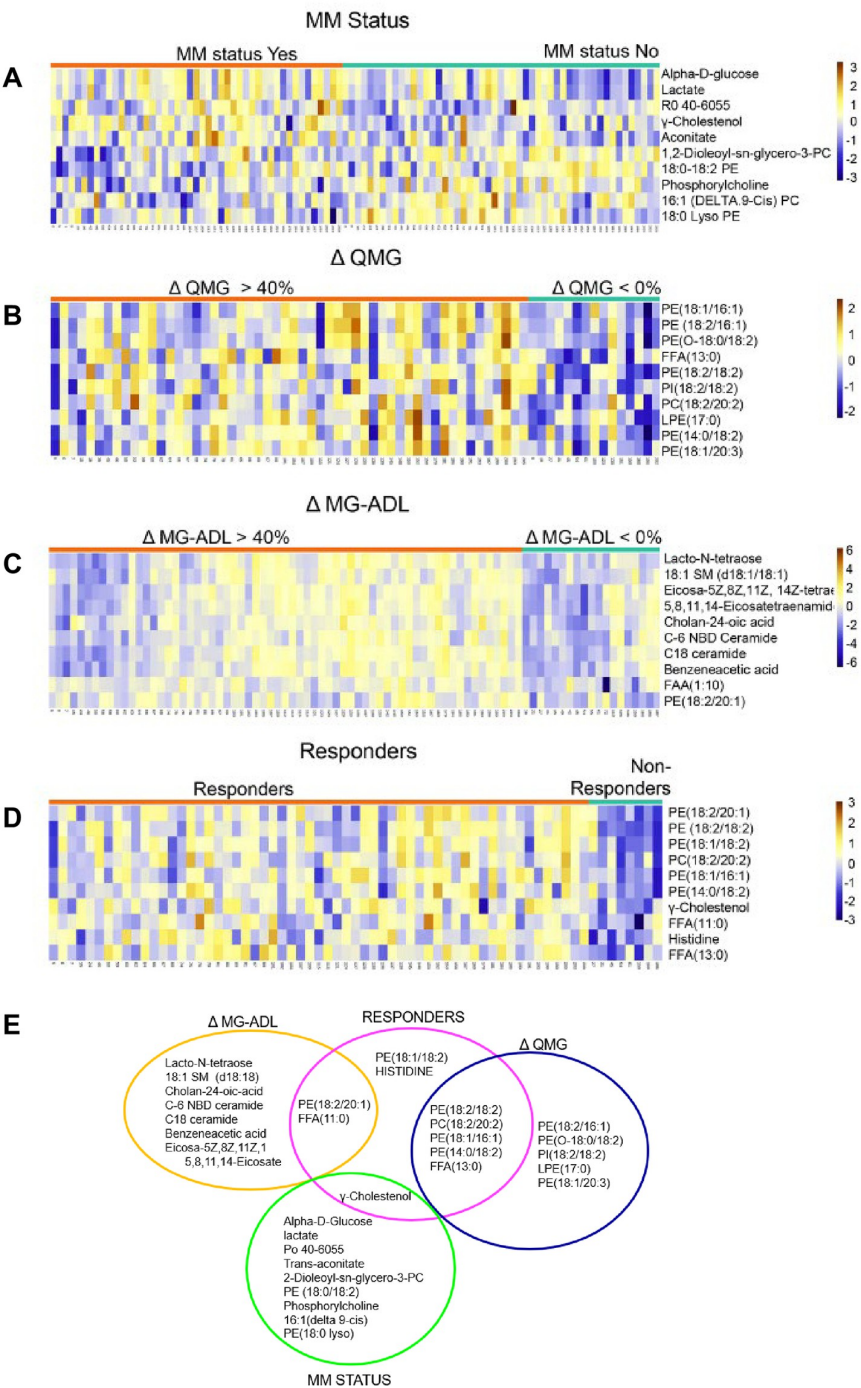
Top 10 significantly changed metabolites and lipids in each clinical outcome measure group. Heatmaps are show in A-D. Each row represents a metabolite or lipid and each column represents a sample. Samples were separated based on clinical outcome (X-axis, orange treatment responsive, teal treatment non-responsive). Each feature is expressed as relative abundance represented in the orange-blue color scale. The orange color indicates increase in levels and the blue color represent decrease in levels. (A) MMS status, yes vs. no (B) change in (Δ) QMG (> 40% vs. < 0%) (C) change in (Δ) MG-ADL (> 40% vs. < 0%), (D) Responder versus Non-Responder. (E) Venn diagram of top 10 altered metabolites and lipids in each outcome group. Differences between outcome groups were tested using the non-parametric Mann Whitney U test and FDR correcting for multiple testing with Benjamini-Hochberg procedure two-sided. P-values < 0.05 are statistically significant. Individual p-values for each metabolite and lipid are reported in S2 Table.

We used hierarchical clustering to visualize the top ten metabolites/lipids as a heat map that provides an overview of differences correlating with outcome measures (Fig 5). The heat maps show dichotomous stratification of metabolites based on MMS (Fig 5A), change in QMG (Fig 5B), change in MG-ADL (Fig 5C), and Responders classification (Fig 5D). There are general trends of greater relative abundance (red color) of several classes of lipids across responders, change in QMG > 40% and MG-ADL > 40% groups, whereas non-responders and those with no change or worsening QMG and MG-ADL scores showed significant lower concentrations (blue color). MMS map on visual inspection shows the greatest variation among individuals in metabolite/lipid expression.

This analysis enabled us to demonstrate differences in the serum metabolite abundances based on outcome measures. QMG (Fig 5B) and Responders (Fig 5D) categorization identifies low levels of phosphatidylethanolamines (PE) at study entry in the subjects with a poor response. PEs are glycerophospholipids with different combinations of fatty acids of varying lengths (16, 18, 20 carbons) and are integral components of biological membranes. The greatest overlap in the top 10 metabolites/lipids occurs between QMG and Responders group (Fig 5E). At least one metabolite in Responders is in common with QMG, MG-ADL and MMS, while no overlap between MG-ADL, MMS and QMG groups is observed. Several lipids and one FFA found in the delta QMG group were also identified in the Responder group, which include PE (18:2/18:2), PE (18:1/16:1), PC (18:2/20:2), and FFA (13:0). There was little concordance in metabolites or lipids in the MG-ADL group compared to the other clinical outcome measures. Lacto-N-tetraose was reduced in poor responders by MG-ADL. This glycosphingolipid binds galectins, which in circulation have been identified to be markers of severity of inflammatory bowel disease and systemic lupus [23, 24]. Free fatty acid (11:0) and PE (18:2/20:1) were common to both MG-ADL and Responder groups. y-cholestenol was elevated in responders in the MMS group and the Responder classification. Overall, the subjects with poor treatment response regardless of outcome measure had reduced levels of several metabolites and lipids at study entrance compared to those that improved (Fig 5).

### Serum metabolomic profiles associated with thymectomy

In order to assess for potential mechanisms of the efficacy of thymectomy, we analyzed metabolites and lipids at 6 months after thymectomy and their change in abundance form baseline and 6 months. We did not find any relationship to having a thymectomy by either analysis (S1 Fig and S3 Table).

### Evaluation of biomarker efficacy

**Fig 6:**
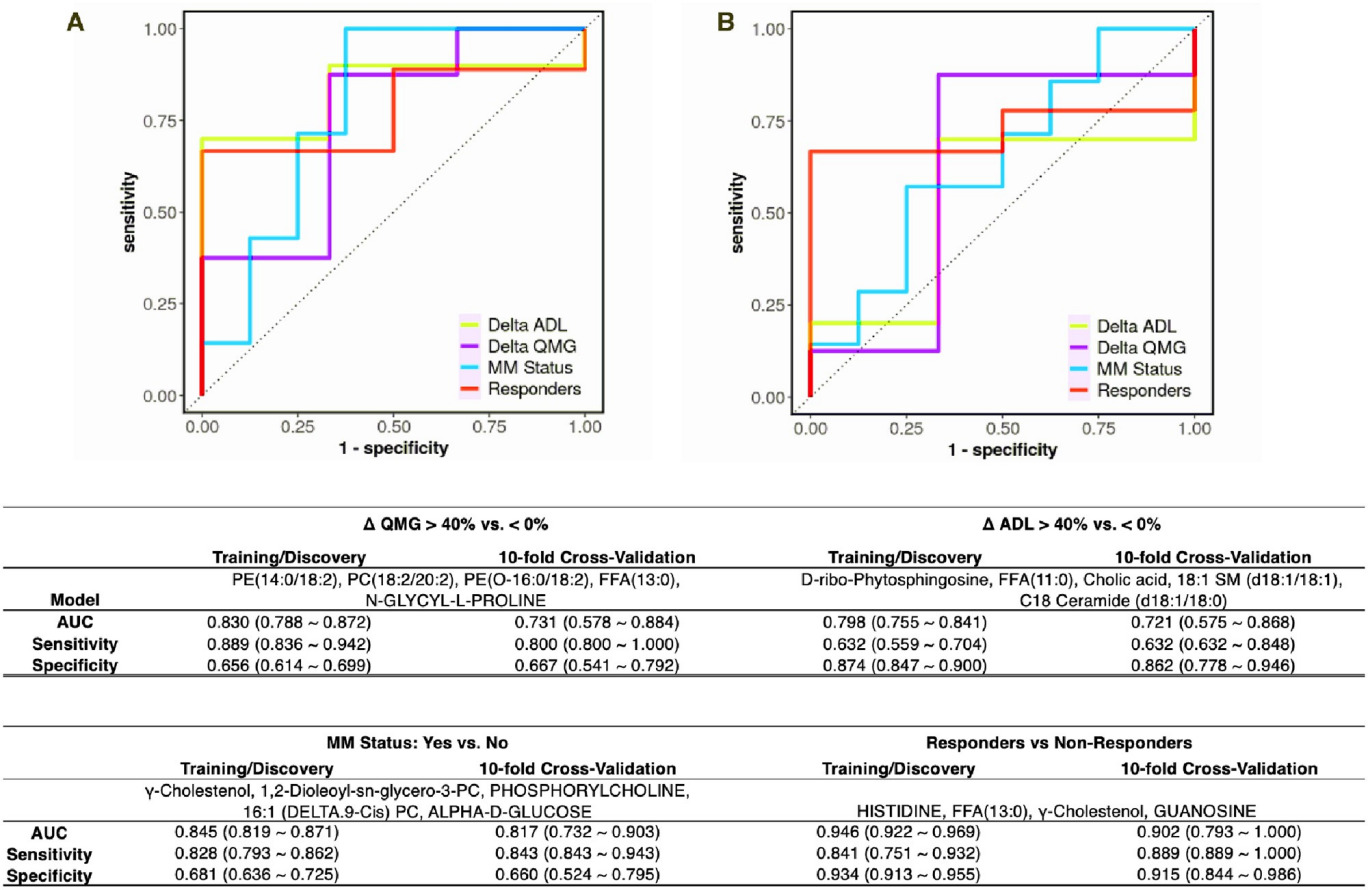
ROC curve analysis for predictive biomarkers. ROC curve analysis (sensitivity on y-axis and specificity on x-axis) for all subjects (A) training set to test performance and (B) validation set. ROC curve for MG-ADL (delta ADL) is represented in yellow; QMG (delta QMG) in purple; MM status in blue; and Responders in orange. Table shows values for area under the curve, sensitivity, and specificity.

To assess potential prognostic value of these metabolic signatures, we used the LASSO (Absolute Shrinkage and Selection Operator) regression algorithm to select features (metabolites and lipids) that correlated with treatment response (Fig 6). To ensure independence between training and testing sets and avoid over fitting, 10-fold cross-validation was performed that yielded a 4–5 panel of metabolites for each clinical outcome group. These panels were used to construct receiver operating characteristic (ROC) curves such that addition of more metabolites/lipids did not enhance predictive performance. The panel of metabolites predictive of distinguishing between Responders and Non-responders, consisting of Histidine, FFA(13:0), γ-Cholestenol and guanosine yielded an area under the curve (AUC) of 0.902 with a specificity of 91.5% and sensitivity of 88.9%. The other outcome measures of QMG, MG-ADL, and MMS gave lower AUCs (0.731, 0.721 and 0.817 respectively) (Fig 6A and 6B). Together these results highlight the utility of metabolomics-based approaches in identifying predictive features of treatment response in MG. We repeated the ROC analysis after removal of subjects who had not received prednisone at study entrance (S2 Fig). Using the same prediction panel, the AUC for ROC slightly decreased for QMG, MG-ADL, and MMS (0.724, 0.722, and 0.752, respectively). The AUC in Responders’ prediction after removal of no prednisone subjects did not change (0.900), however sensitivity was increased (93.5%). Further, we assessed whether histidine, FFA(13:0), γ-cholestenol, and guanosine separately associated with age and stratified also by gender and ethnicity/race (S3A and S3D Fig). Pearson’s correlation’s R-value and p-value were calculated for each comparison. None of variables showed significant correlations indicating that race, age, and gender did not have a major effect on serum metabolites predicting outcome.

### Pathway analysis

**Fig 7:**
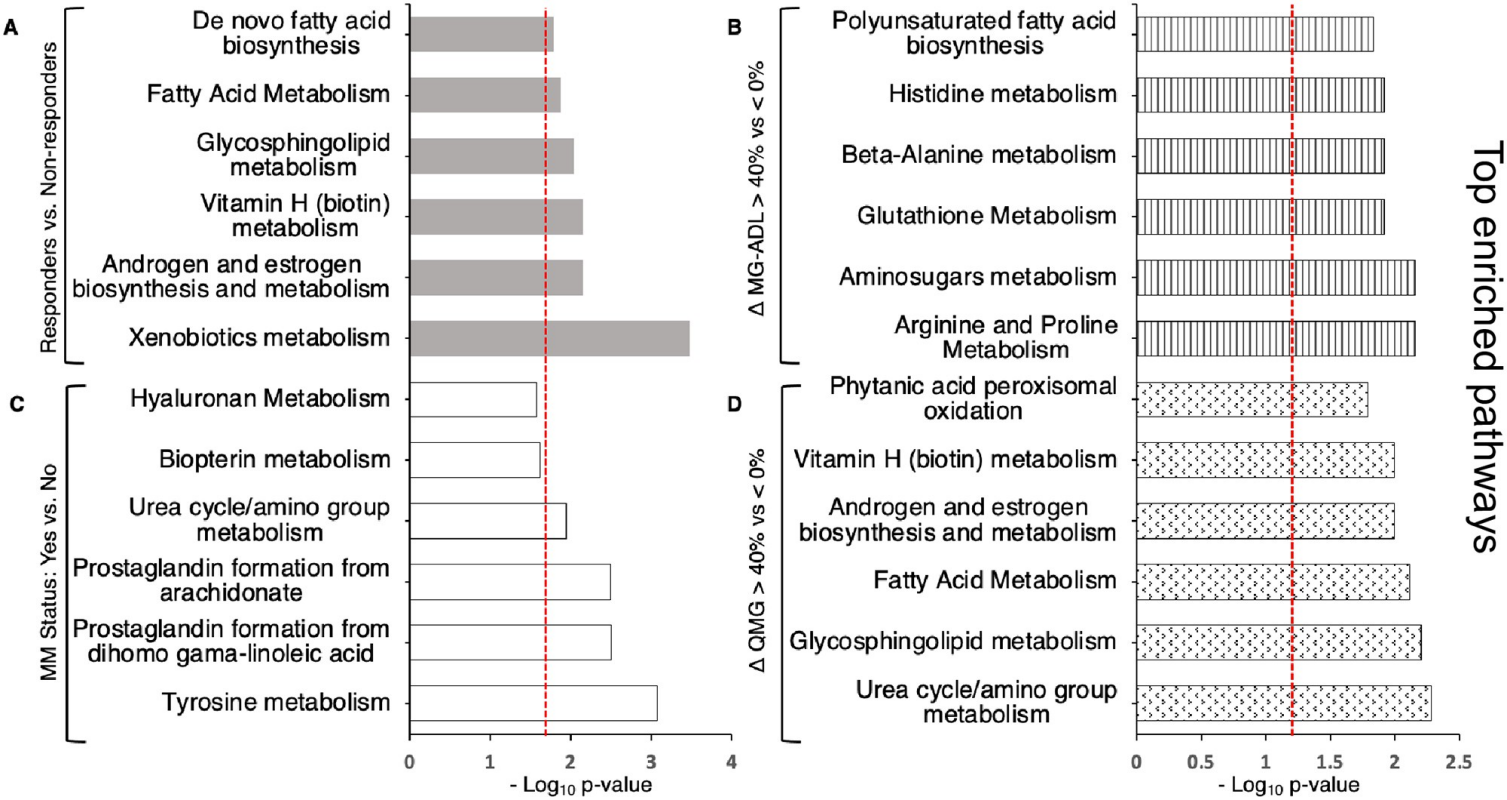
Metabolic pathway analysis for four outcome measure. Top significantly enriched pathways (treatment responsive vs treatment non-responsive) in each outcome measure: (A) Responders (gray shaded bars), (B) change in (Δ) MG-ADL score (vertical stripped bars), (C) MMS (open bars), and (D) change in (Δ) QMG score (dotted pattern bars). Pathway enrichment was performed using *mummichog* (Fisher’s exact test). P-values are shown on x-axis and specific values are reported in S4 Table.

We performed pathway analysis on the metabolic and lipidomic profiles for each outcome group using the Mummichog package to gain biological insight into underlying metabolic pathways associated with responsiveness to treatment [25]. Fig 7 shows the top six pathways significantly altered in each outcome measure, which largely parallels the trends observed in the metabolic and lipidomic profiles for each outcome group. Like the univariate analysis (Fig 4), metabolic pathways associated with MG-ADL and MMS stand out as the most distinct. A total of 9 out of 12 pathways are significantly altered between those with improved MG-ADL scores and those that did not improve or worsened (S4 Table). These pathways include those related to protein metabolism (arginine and proline metabolism, amino sugar metabolism, histidine metabolism, beta-alanine metabolism), and glutathione metabolism. Polyunsaturated fatty acid biosynthesis and aspartate and asparagine metabolism pathways are common to both the MG-ADL and QMG groups, whereas fatty acid metabolism is common to MG-ADL, QMG, and Responders group. Like the MG-ADL group, 7 of 10 altered pathways are unique to MMS alone. The pathways with the most significant p values associate with tyrosine metabolism and prostaglandin formation pathways (Fig 7), both of which are important mediators in inflammatory responses. Alterations in catabolic pathways such as urea cycle/amino group metabolism, were common to both MMS and QMG groups, and phytanic acid peroxisomal oxidation was common to MMS, QMG and Responders groups.

The greatest agreement between groups in the pathway analysis is between the QMG group and Responders group. A total of seven pathways related to glycosphingolipid metabolism, Vitamin H (biotin) metabolism, fatty acid metabolism, and androgen and estrogen biosynthesis and metabolism, polyunsaturated fatty acid biosynthesis, aspartate and asparagine metabolism, and C21-steroid hormone biosynthesis are commonly altered in these two outcome groups. Interestingly, the most significant p value in the Responders group is associated with xenobiotic metabolism, which includes drug metabolism.

## Discussion

This analysis has identified several important biosynthetic and catabolic pathways that are differentially expressed between treatment-responsive and treatment-resistant patients from the MGTX trial. Several shared pathways related to protein metabolism and lipid metabolism are altered across most outcome measures, however the physician-determined MMS and patient-reported MG-ADL groups were found to associate with distinct pathways. These results suggest that differences in patient-reported versus observer-reported outcomes contribute to these discrepancies. Together, our findings suggest reduced levels of certain metabolites and lipids at baseline may predict poor responses to prednisone treatment. How specific alterations in these pathways might contribute to corticosteroid resistance remains to be elucidated. Implementation of predictive models at 6 months will further distinguish between prednisone-responsive versus thymectomy-responsive biomarkers.

### Metabolite panel predicts treatment response

We took an unbiased metabolomic discovery approach which identified a panel of four serum metabolites at time of entry to the MGTX trial, which were highly predictive of a strictly defined outcome of treatment response at six months of standard-of-care therapy for MG (sensitivity 0.89, specificity 0.91). One purpose of the investigation was to perform a discovery study of prednisone treatment regardless of prior treatment, which is a typical clinical situation. Slightly more than three quarters of subjects were on some dose of prednisone at study entry. Removal of subjects not on prednisone did not significantly alter AUCs and only enhanced the sensitivity of detection of severely treatment resistant subjects. Histidine, FFA(13:0), γ-Cholestenol and guanosine as a group were highly predictive of a rigorous treatment response. Serum histidine has been found to be predictive of treatment-response to PD-1 inhibitors, which modify the immune response to small cell cancer [26]. The investigators related this effect to variations in amino acid metabolism and T-cell transport in cancers exposed to PD-1 inhibition. A similar explanation could be present in MG given similarities between neoplastic and autoreactive cells [27]. Cholestanol is an intermediate of bile acid synthesis that derives from the catabolism of cholesterol [28]. Little is known about the normal function of cholestanol, but pathological accumulation of cholestenol enhances apoptosis. Guanosine is known to have a significant immunomodulatory influence on nervous system pathology [29], but systemic effects are less well defined.

### Metabolomic signatures vary based on clinical outcome measure

Clinical outcome measures used in the MGTX study assess a patient’s status from their own perspective (MG-ADL) an examiner’s assessment of muscle strength at a given time (QMG), and a snapshot of a physician’s global impression of the patient (MMS). Several studies have demonstrated that these measures correlate only in a moderate fashion indicating that they are assessing varying and non-overlapping aspects of the disease [16, 30, 31]. Our results are consistent with these measures and show that each outcome measure was associated with distinct metabolomic and lipidomic signatures. The MG-ADL was the greatest outlier, suggesting fundamentally different biological underpinnings compared to other outcome measures. The volcano plot analysis shows that MMS, change in QMG, and the Response category share significant elevations in PE, but the change in MG-ADL does not. MMS is physician determined and dependent on the combined assessment of patient symptoms and a physical examination at a point in time and thereby shares features with the QMG. In contrast, the MG-ADL is purely a patient assessment of ability to perform specific activities in the days prior to assessment. Similar trends were demonstrated in the pathway analysis, with the Responders and QMG groups sharing a majority of altered pathways with one or more outcome groups, whereas the pathway analysis of the MG-ADL was again distinct. The results of this study have highlighted the importance of defining outcome measures for MG in discovery phase biomarker studies.

### Metabolic pathways associated with treatment response

All subjects in the MGTX study were on a standardized protocol of prednisone treatment at study entry. The alterations in metabolic pathways observed for treatment responsive subjects are consistent with the well-established effects of corticosteroids on protein metabolism, fatty acid metabolism, and de novo lipid biosynthesis [32–35]. We also observed alterations in these pathways in our previous metabolomic study, which compared the metabolome of MG subjects before and after receiving twelve weeks of 20 mg of prednisone [36]. Lipids were particularly associated with treatment response, as readily appreciated by the volcano plot analysis. Increased abundance of several ceramides, the shortest of the sphingolipids, was observed for the MG-ADL group (Fig 4). Significant alterations in glycosphingolipid (GLP) metabolism, demonstrated by increased abundance of PEs found at baseline, were observed in both the QMG and Responder groups. Phospholipids are integral components of the plasma membrane and play an important role in regulating immune cell function [37]. Lipid metabolism is increasingly being recognized as an important factor influencing immune cell differentiation and activation [38]. Changes in lipid profiles or metabolism can thus impact cellular signaling involved in inflammatory responses [39]. Dysregulated GLP expression in CD4+ T cells from SLE patients has been associated with T cells dysfunction [40]. In addition to influencing inflammatory pathways, certain lipids also impact emotional states, which have the potential to impact patient reported outcomes [39].

Corticosteroid treatment is known to induce protein catabolism in healthy adults and patients with SLE leading to increased plasma levels of amino acids [41, 42]. Perturbations in protein metabolism observed in our study may reflect responsiveness to corticosteroid treatment in MG for those subjects on prednisone at baseline. Urea cycle and amino group metabolism were among the most significantly enriched pathways for both MMS and change in QMG outcome measures. Several amino acid metabolism pathways were also significantly enriched in the change in MG-ADL group (arginine and proline, amino sugar, glutathione, beta-alanine, and histidine metabolism). The observed enrichment of amino acid biosynthetic pathways at baseline for treatment responsive subjects may suggest a potential increase in demand for amino acids and increase in urea cycle pathways to deal with protein catabolism. Urine metabolomic profiling studies showed that increases in amino acid levels in urine were sustained on day 15 of high dose (30 mg) prednisolone [33]. Earlier studies comparing acute and chronic corticosteroid use indicated protein oxidation stabilized with chronic use, suggesting metabolic adaptation occurs to limit protein loss [43].

Glycosphingolipid (GLP) metabolism, urea cycle, and pentose phosphate pathways are associated with *in vitro* glucocorticoid resistance [44], and *in vitro* lymphocyte sensitivity to GC have been found to be predictive of treatment response [45–48]. Interestingly, differences in *in vitro* lymphocyte sensitivity occur across ethnic groups to glucocorticoids, which reflect alterations in transcriptional regulation of glucocorticoid responsive genes. These variations could also be reflected in differences in circulating metabolic profiles of treatment-resistant patients. For MG, polymorphisms in the glucocorticoid receptor associate with treatment response in MG [49]. Future studies will assess glucocorticoid receptor expression and *in vitro* sensitivity of PBMCs to corticosteroid treatment in treatment unresponsive and treatment responsive subjects.

Several fatty acid and amino acid metabolites were identified as putative biomarkers in patients with congenital adrenal hyperplasia that were significantly altered in low versus high glucocorticoid dose studies [50]. Further, a recent multi-omic analysis identified several transcriptional and metabolomic predictive markers of glucocorticoid activity from patient blood samples, which included amino acid metabolites and the pyrimidine metabolite uracil [51]. Contrarily, plasma metabolites compared before and after seven weeks of prednisone in children with pediatric nephrotic syndrome poorly responsive to corticosteroids showed lower degrees of metabolite alterations compared to treatment responsive children [52]. 2-Dioleoyl-sn-glycero-3-PC was the only metabolite predictive of steroid resistance, which we identified as a predictive biomarker in the MMS group. In our strictly defined outcome measure of Non-Responders, all subjects were on prednisone at study entry. We speculate that low abundance of specific metabolites and lipids suggest the absence of specific glucocorticoid-mediated changes in non-responsive patients and may be reflective of corticosteroid resistance due to alterations in the metabolism or biological targets of prednisone [53].

### Xenobiotic metabolism may mediate differences in treatment response

The pathway most associated with the stringently defined patient outcome group (Responders) was xenobiotic metabolism. Urine metabolomic profiling of healthy volunteers also demonstrated alterations in xenobiotics in subjects treated with high dose prednisone [33]. Interactions occur between glucocorticoid effects on drug metabolism and the xenobiotic pathways that metabolize prednisone [54]. Both nuclear steroid and xenobiotic receptor activation inhibit the activity of NF-κB, a master regulator of the immune response [55]. NF-κB activation also reciprocally inhibits xenobiotic metabolism, creating a complex feedback loop [55]. Differences in transcription of NF-κB and other genes are associated with the degree of lymphocyte sensitivity to glucocorticoids [45, 56]. Therefore, a major contributor to response to prednisone may be related to variations in metabolism of the drug across individuals.

### Limitations

Inherent to all rare disease research is the significant challenge to recruit a cohort of rigorously clinically characterized and monitored patients coupled to biospecimen collection and uniform treatment protocols. To date only the MGTX study fulfills all these requirements, and we took advantage of the biospecimen bank to perform a discovery study, which will require validation. The value of metabolomics and lipidomic analysis as an unbiased assessment for biomarker discovery and characterization of disease mechanisms cannot be questioned. Since the goal was to find lipids and metabolites that are treatment-predictive specific to MG patients, determining a profile specific to the disease itself is not relevant. In fact, we suspect the steroid-treatment response will be shared by other autoimmune disorders. There are numerous factors impacting the metabolome which are impossible to fully account for in any investigation. It is possible that improved strength will lead to increased activity [57], alteration in diet, and improved emotional state, each of which could impact the metabolic profile [58]. However, we would argue that these factors contribute to the treatment-response and that they do not reduce the significance of our results. We found markers predictive of treatment response in a reasonable sample size for each of the outcome measures with AUC estimates above 0.7.

## Conclusion

As corticosteroid resistance is a common phenomenon across autoimmune and inflammatory diseases, these findings also have potential broad relevance to predicting treatment responses across other conditions. This discovery would be the first identification of potentially predictive biomarkers for clinical outcome in MG. An independent validation cohort, however, is needed to confirm this panel as treatment-predictive. The metabolites and lipids are involved in immunologic and regulatory pathways providing a likely mechanistic link to treatment failure. In immune cells, non-genomic interaction of GCs results in biophysical changes in the plasma membrane that are believed to impact the function of membrane associated proteins and calcium and sodium cycling, which may also contribute to rapid immunosuppressive and anti-inflammatory effects [59–61]. Such membrane mechanics are likely to influence corticosteroid treatment response in an individual. Greater understanding of these mechanisms will enhance personalized treatment approaches to better predict favorable and adverse effects of prednisone on the individual.

## Supporting information

S1 Fig Volcano plot showing differentially abundant serum metabolites at 6 months versus baseline for thymectomy + prednisone group. Gray dots represent metabolites and lipids that were not significantly altered in thymectomy + prednisone group at 6 months versus baseline. Purple dots represent metabolites with a fold change greater than 2-fold (cutoff), orange dots represent metabolites and lipids with a significant p value (p < 0.05) and green dots indicates those metabolites and lipids with both a significant fold change and p-value. Differences were tested using the non-parametric Mann Whitney U test and FDR correcting for multiple testing with Benjamini-Hochberg procedure two-sided. Individual p-values for each metabolite and lipid are found in S3 Table.
(DOCX)

S2 Fig ROC curve analysis of predictive biomarker panels for each outcome group after removal of subjects not on prednisone at study entry. (A) MM Status, (B) Responders (C) change in (Δ) QMG score (>40% vs ≤ 0%) and (D) change in (Δ) MG-ADL score (>40% vs ≤ 0%).
(DOCX)

S3 Fig No major effects of race, age, and gender on serum metabolomic and lipidomic profiles. Age was plotted against Histidine, FFA(13:0), γ-Cholestenol, and guanosine, separately. Plots were also stratified by Gender and Race. Pearson’s correlation’s R-value and p-value were calculated for each. No significant correlations were observed between the metabolite and age, or gender and race.
(DOCX)

S1 Table Tandem MS validated metabolites. List of annotated metabolites and lipids.
(XLSX)

S2 Table Test statistics for targeted and unvalidated targeted metabolites. Group differences were tested using the non-parametric Mann Whitney U test and FDR correcting for multiple testing with Benjamini-Hochberg procedure. P-values < 0.05 were regarded as statistically significant.
(XLSX)

S3 Table Thymectomy + prednisone 6 months vs. baseline. Metabolites and lipids at 6 months after thymectomy and the change in abundance from baseline and 6 months.
(XLSX)

S4 Table Mummichog pathway analysis. (XLSX)
