# Supplementary material for: Serum metabolomics of treatment response in myasthenia gravis
Source: PLoS One. 2023 Oct 10;18(10):e0287654. doi: 10.1371/journal.pone.0287654 (PMC10564178; doi:10.1371/journal.pone.0287654)
Supplement: S1 Fig — Gray dots represent metabolites and lipids that were not significantly altered in thymectomy + prednisone group at 6 months versus baseline. Purple dots represent metabolites with a fold change greater than 2-fold (cutoff), orange dots represent metabolites and lipids with a significant p value (p < 0.05) and green dots indicates those metabolites and lipids with both a significant fold change and p-value. Differences were tested using the non-parametric Mann Whitney U test and FDR correcting for multiple testing with Benjamini-Hochberg procedure two-sided. Individual p-values for each metabolite and lipid are found in S3 Table. (DOCX) [file pone.0287654.s001.docx]

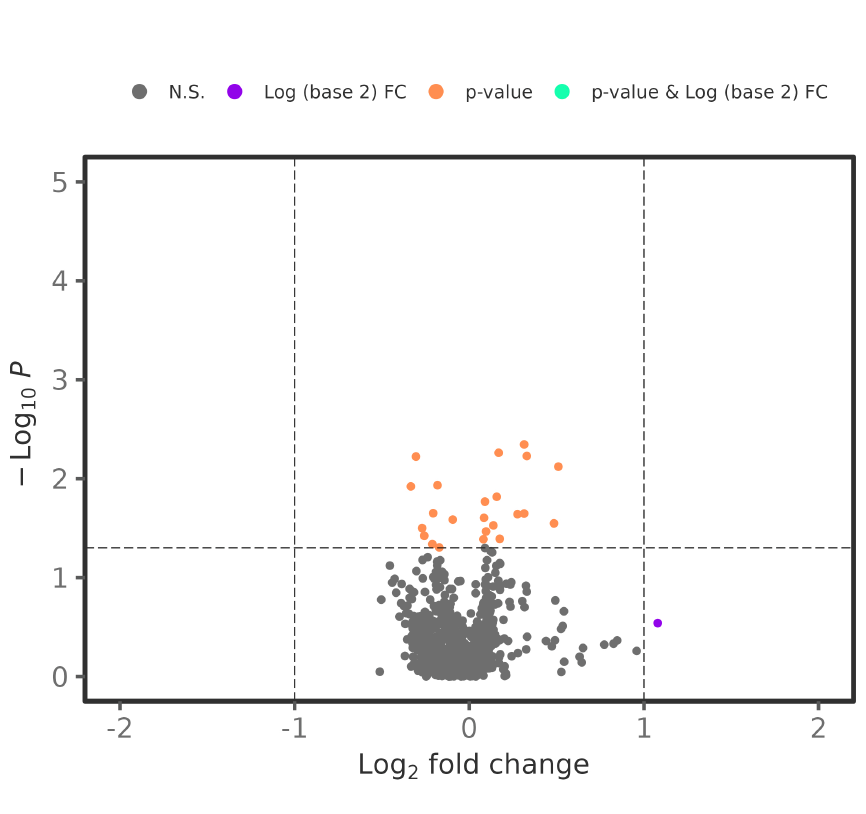


**S1 Fig. Volcano plot showing differentially abundant serum metabolites at 6 months versus baseline for thymectomy + prednisone group.** Gray dots represent metabolites and lipids that were not significantly altered in thymectomy + prednisone group at 6 months versus baseline. Purple dots represent metabolites with a fold change greater than 2-fold (cutoff), orange dots represent metabolites and lipids with a significant p value (p < 0.05) and green dots indicates those metabolites and lipids with both a significant fold change and p-value. Differences were tested using the non-parametric Mann Whitney U test and FDR correcting for multiple testing with Benjamini-Hochberg procedure two-sided. Individual p-values for each metabolite and lipid are found in S3 Table.
