# Supplementary material for: Serum metabolomics of treatment response in myasthenia gravis
Source: PLoS One. 2023 Oct 10;18(10):e0287654. doi: 10.1371/journal.pone.0287654 (PMC10564178; doi:10.1371/journal.pone.0287654)
Supplement: S2 Fig — (A) MM Status, (B) Responders (C) change in (Δ) QMG score (>40% vs ≤ 0%) and (D) change in (Δ) MG-ADL score (>40% vs ≤ 0%). (DOCX) [file pone.0287654.s002.docx]

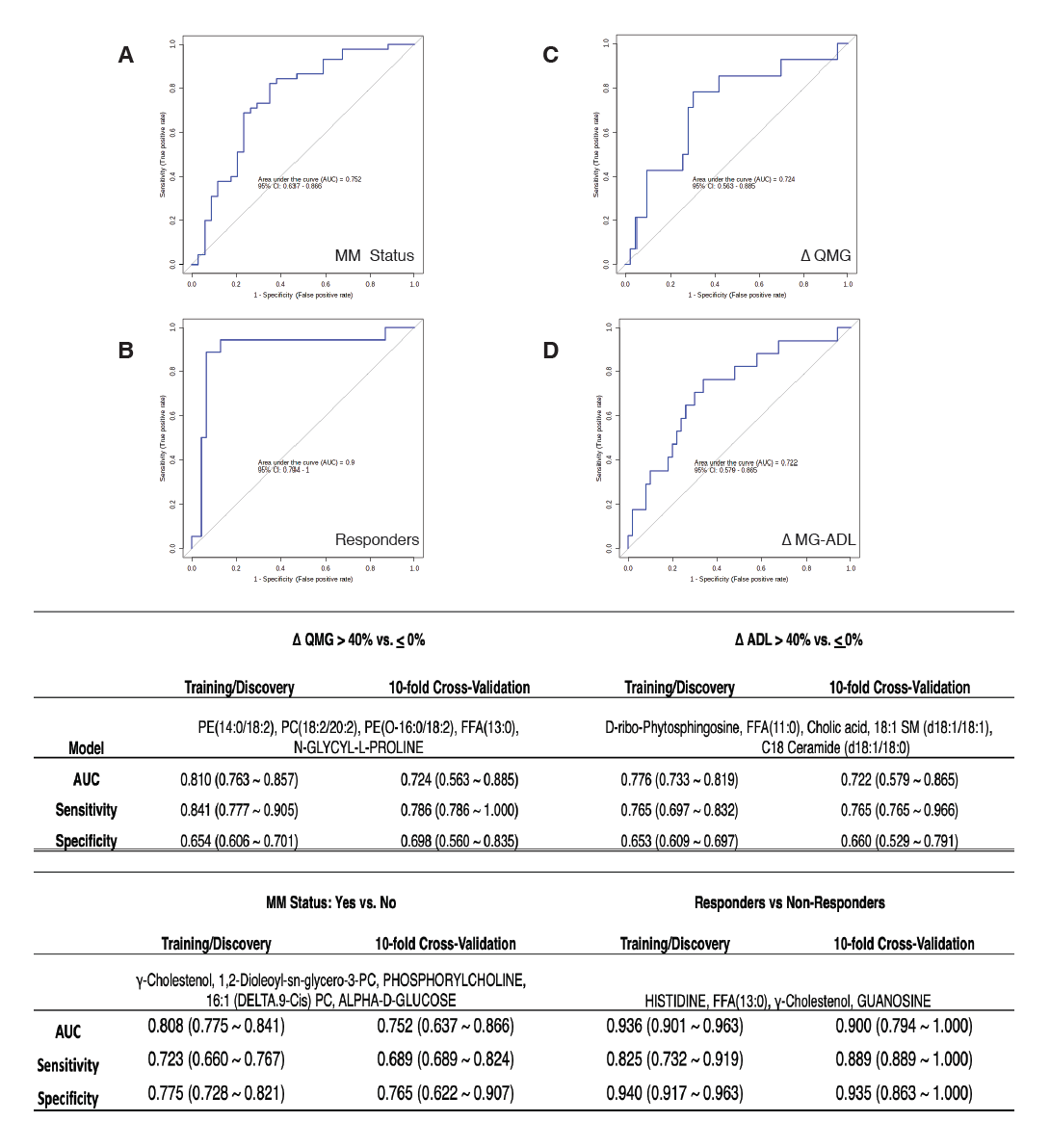


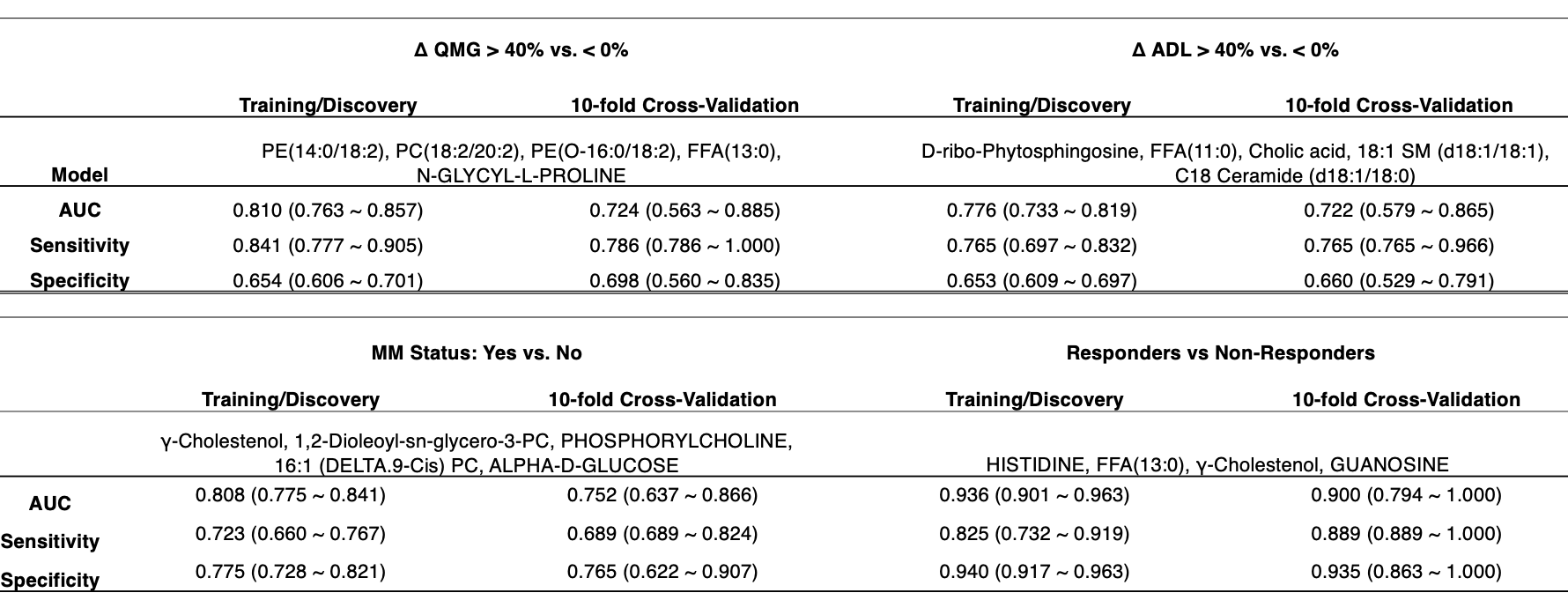


**S2 Fig.** **ROC curve analysis of predictive biomarker panels for each outcome group after removal of subjects not on prednisone at study entry.** (A) MM Status, (B) Responders (C) change in (∆) QMG score (>40% vs < 0%) and (D) change in (∆) MG-ADL score (>40% vs < 0%).
