# Supplementary material for: Serum metabolomics of treatment response in myasthenia gravis
Source: PLoS One. 2023 Oct 10;18(10):e0287654. doi: 10.1371/journal.pone.0287654 (PMC10564178; doi:10.1371/journal.pone.0287654)
Supplement: S3 Fig — Age was plotted against Histidine, FFA(13:0), γ-Cholestenol, and guanosine, separately. Plots were also stratified by Gender and Race. Pearson’s correlation’s R-value and p-value were calculated for each. No significant correlations were observed between the metabolite and age, or gender and race. (DOCX) [file pone.0287654.s003.docx]

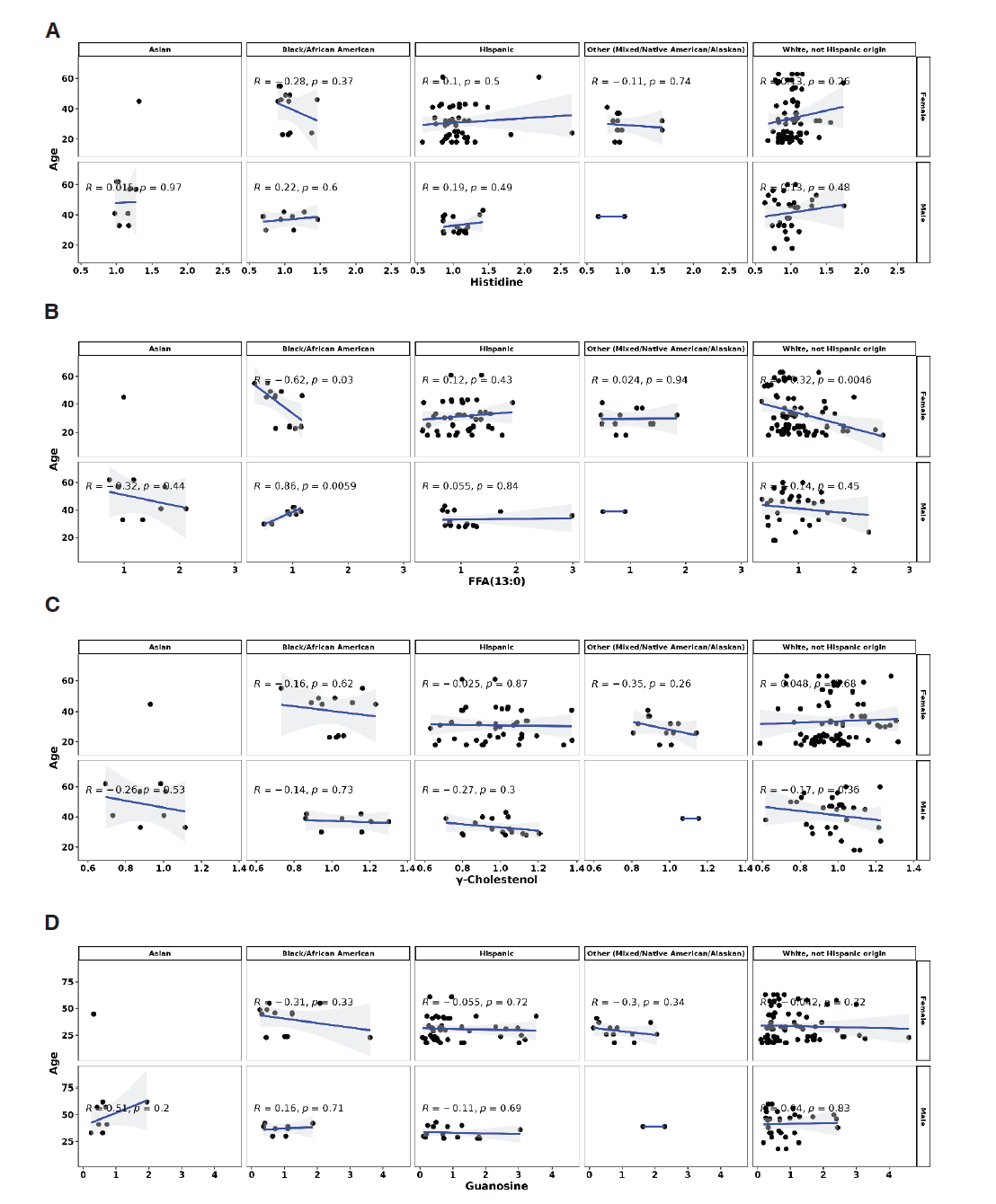


**S3 Fig. No major effects of race, age, and gender on serum metabolomic and lipidomic profiles.** Age was plotted against Histidine, FFA(13:0), γ-Cholestenol, and guanosine, separately. Plots were also stratified by Gender and Race. Pearson’s correlation’s R-value and p-value were calculated for each. No significant correlations were observed between the metabolite and age, or gender and race.
